# Biomimetic Visual Information Spatiotemporal Encoding Method for In Vitro Biological Neural Networks

**DOI:** 10.3390/biomimetics10060359

**Published:** 2025-06-03

**Authors:** Xingchen Wang, Bo Lv, Fengzhen Tang, Yukai Wang, Bin Liu, Lianqing Liu

**Affiliations:** 1 State Key Laboratory of Robotics, Shenyang Institute of Automation, Chinese Academy of Sciences, Nanta Street 114, Shengyang 110016, China; wangxingchen231@mails.ucas.ac.cn (X.W.); liubin@sia.cn (B.L.); lqliu@sia.cn (L.L.); 2 University of Chinese Academy of Sciences, Beijing 100049, China; 3 Key Laboratory of Organ Regeneration and Reconstruction, Institute of Zoology, Chinese Academy of Sciences, Beijing 100101, China; wangyukai@ioz.ac.cn; 4 Beijing Institute for Stem Cell and Regenerative Medicine, Beijing 100101, China

**Keywords:** in vitro biological neural network, high-density microelectrode arrays, visual information encoding, neural activity decoding

## Abstract

The integration of in vitro biological neural networks (BNNs) with robotic systems to explore their information processing and adaptive learning in practical tasks has gained significant attention in the fields of neuroscience and robotics. However, existing BNN-based robotic systems cannot perceive the visual environment due to the inefficiency of sensory information encoding methods. In this study, we propose a biomimetic visual information spatiotemporal encoding method based on improved delayed phase encoding. This method transforms high-dimensional images into a series of pulse sequences through convolution, temporal delay, alignment, and compression for BNN stimuli. We conduct three stages of unsupervised training on in vitro BNNs using high-density microelectrode arrays (HD-MEAs) to validate the potential of the proposed encoding method for image recognition tasks. The neural activity is decoded via a logistic regression model. The experimental results show that the firing patterns of BNNs with different spatiotemporal stimuli are highly separable in the feature space. After the third training stage, the image recognition accuracy reaches 80.33% ± 7.94%, which is 13.64% higher than that of the first training stage. Meanwhile, the BNNs exhibit significant increases in the connection number, connection strength, and inter-module participation coefficient after unsupervised training. These results demonstrate that the proposed method significantly enhances the functional connectivity and cross-module information exchange in BNNs.

## 1. Introduction

The biological neural network (BNN) is a complex, multi-layered network system composed of numerous interconnected neurons, which processes information through synaptic transmission and demonstrates exceptional computational properties [1,2,3]. Research indicates that in vitro BNNs can self-organize into specific structural and functional connections, displaying biological intelligence comparable to that of the in vivo brain [4,5,6]. Exploiting these properties, researchers have integrated in vitro BNNs with robotic systems to overcome the limitations of invasive studies and enable direct interaction between BNNs and external environments [7,8]. Furthermore, growing in vitro BNNs on high-density multielectrode arrays (HD-MEAs) allows for multi-channel electrical stimulation and the high-resolution recording of the network activity [9,10]. The development of this technology facilitates the deeper integration of in vitro BNNs with robotic systems. Robotic systems based on in vitro BNNs aim to combine the benefits of biological intelligence with electromechanical systems to carry out tasks involving perception, cognition, and decision-making [11,12,13]. Therefore, they are garnering increasing interest in both neuroscience and robotics [8].

In BNN-based robotic systems, in vitro BNNs are unable to process external environmental information directly. Therefore, they must be trained using neural signal encoding and decoding methods to develop specific functions. Neural signal encoding translates environmental inputs into spatiotemporal stimulation patterns, while decoding transforms network activity into interpretable control signals [14]. The current in vitro BNN implementations predominantly employ linear encoding, binary encoding, spatial encoding, or spatiotemporal combined encoding strategies, coupled with proportional mapping, binary mapping, and machine learning decoding approaches. Early studies primarily focused on elementary behavioral control using in vitro BNNs by employing simplified encoding and decoding frameworks. In mobile robotics studies on obstacle avoidance or tracking, one-dimensional sensor data have been transduced via linear encoding [15,16,17], binary encoding [18,19,20], or spatial encoding [21] into pulse stimuli. The wheel differential control is then achieved through either firing-rate proportional mapping [15,16,17] or threshold-based binary mapping decoding [18,19,20,21]. More recently, in vitro BNNs have been increasingly applied to more complicated tasks demanding adaptive learning capabilities. Kagan et al. [22] integrated linear encoding and spatial encoding methods to employ in vitro BNNs within an arcade game, ’Pong’, utilizing time-division stimulation across multiple electrodes to encode the one-dimensional positional data of the ball relative to the paddle. However, their study employed a binary mapping decoding method, which was only capable of achieving the vertical movement of the paddle. Cai et al. [23] developed a novel combined encoding framework integrating temporal encoding with spatial encoding. By incorporating temporal delays into multi-channel pulse sequences encoding speech signals and chaotic equations, coupled with linear classifiers and regression decoders, they achieved speech recognition and nonlinear sequence prediction. Their study shows that combined encoding is markedly superior to singular encoding approaches. Ades et al. [24] implemented the linear encoding of tactile feedback for a robotic hand, with a binary mapping decoding strategy based on threshold detection to govern individual joint actuation. A systematic review reveals that the constraints of existing encoding methods limit in vitro BNNs to one-dimensional sensor inputs, rendering them ineffective in processing high-dimensional visual data. Moreover, such constraints impede their applicability in intricate visual tasks like image recognition, a capability that is crucial for environmental perception, object detection, and higher-level decision-making. A novel visual information encoding method is therefore essential to harness BNNs’ latent computational power for real-world vision tasks.

Pulse signals serve as the primary means of communication in in vitro BNNs. To effectively handle information, visual image data from electromechanical systems must be converted into pulse sequences, which are then input into a small subset of neurons. This process is influenced by the biological limitations of cultured BNNs, which include constraints on the population size and stimulation tolerance. These limitations impose fundamental boundaries on the complexity of the encoded pulse sequences required to ensure the network’s viability. Traditional neural-like computing algorithms often employ temporal delay phase encoding and Poisson encoding strategies for the processing of sensory information [25,26]. However, these conventional encoding methods are limited to processing grayscale images and cannot handle high-dimensional color images. Additionally, these methods map each pixel to a pulse, leading to a pulse sequence with a high stimulation frequency and requiring a large number of input neurons. Previous studies have shown that intense high-frequency stimulus inputs can diminish or eliminate the responses of in vitro BNNs and may even lead to network dysfunction [27,28]. Therefore, these methods cannot be directly applied to in vitro BNNs.

In biological visual systems, neurons in the visual cortex do not respond to each individual pixel of an image. Instead, they generate neural spikes by extracting and processing key features of the image, such as edges, motion, color, and shape. Neurons focus on these critical features and employ sparse encoding and selective responsiveness to efficiently process visual information [29,30]. This mechanism enables the brain to handle complex visual inputs without being overwhelmed by the vast amount of redundant pixel data. To emulate this integrated neural pathway, we introduce feature extraction operations into the image encoding process, thereby reducing the complexity of the encoded pulse sequences. Furthermore, spatiotemporal combined encoding shows great potential by utilizing various pulse sequence arrangements and spatial electrode allocation to exploit the neuronal spiking dynamics. This approach generates richer stimulation patterns with an enhanced information capacity [31,32]. Inspired by this, to enable in vitro BNNs to perceive external visual images, the feature-extracted image data can be encoded into spatiotemporal pulse sequences that are then delivered sequentially to the corresponding neurons over time.

The main contributions of this paper can be summarized as follows.

- We propose a biomimetic visual information spatiotemporal encoding method for in vitro BNNs, enabling them to perceive complex visual information effectively. The proposed encoding method first utilizes a convolutional neural network (CNN) to extract features from high-dimensional colored images and then uses a delayed phase encoding scheme to transform the highly compressed features into spatiotemporal pulse sequences that can be accepted by BNNs.
- We propose an unsupervised training process for in vitro BNNs to fulfil image recognition tasks using the proposed encoding scheme and a logistic regression decoding strategy. The images are encoded into pulse sequences and input to the in vitro BNN via an electrical stimulus. The evoked neural network’s activity is decoded into the classes of the images. The in vitro BNN is trained by repetitive stimuli to improve the recognition performance.
- Experimental results show that the image recognition performance of in vitro BNNs is enhanced by this unsupervised training process. A functional connectivity analysis on in vitro BNNs reveals that the trained BNNs show significant improvements in the node degree, node strength, edge weight, and inter-module participation coefficient. These changes indicate the reshaping of the network’s functional structure and enhanced capabilities for cross-module information exchange. These functional connectivity changes may be the main factors that enable the in vitro BNNs to achieve improved performance.

These findings emphasize the potential of BNNs to perform complex tasks through visual perception, while also providing new insights into the learning mechanisms of biological neural networks.

## 2. Materials and Methods

**Figure 1:**
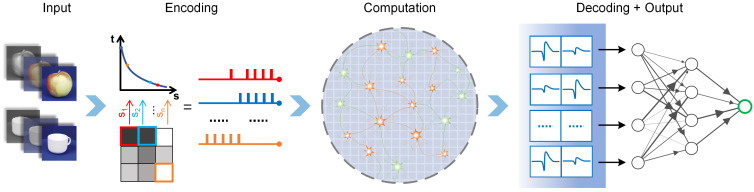
Visual information encoding and decoding framework for in vitro biological neural networks.

The visual information encoding and decoding framework for in vitro BNNs constructed in this study is illustrated in Figure 1. In the encoding phase, high-resolution input images are processed through feature extraction to generate low-resolution feature maps. Specific encoding rules are then applied to convert the pixel intensities of these feature maps into spatiotemporal spike stimulation sequences. These sequences are delivered synchronously to the corresponding neurons, eliciting dynamic activity within the BNNs. The BNNs complete information processing and computation. In the decoding phase, an appropriate decoding algorithm is utilized to extract feature information from the firing patterns of the neural network and output the recognition result. This completes the task of visual image perception and recognition.

### 2.1. In Vitro Biological Neural Network Culture and Signal Acquisition

**Figure 2:**
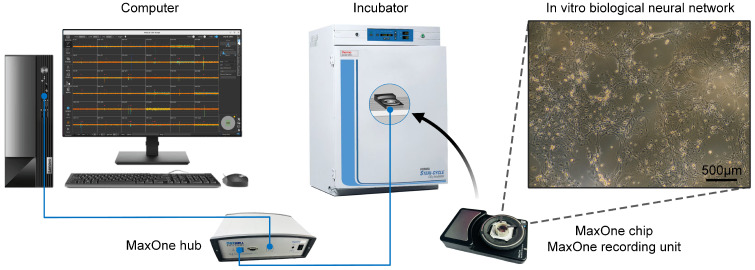
In vitro biological neural network signal acquisition and electrical stimulation input process.

In this study, in vitro BNNs were cultured on high-density microelectrode arrays (HD-MEAs) from MaxOne (see Figure 2). The neurons that formed the in vitro BNNs were hippocampal neurons extracted from Sprague-Dawley rats that were born within 24 h. The neurons were extracted according to a standard procedure, which was reviewed and approved by the Institutional Animal Care and Use Committee of the Institute of Zoology, Chinese Academy of Sciences (IOZ-IACUC-2021-105).

The neurons were cultured as follows. In preparation, MaxOne electrode chips were first coated with 50 µL of 0.07% polyethyleneimine (Sigma-Aldrich, St. Louis, MO, USA) solution for 1 h and then coated with 50 µL of 0.02 mg/mL laminin (Sigma-Aldrich) solution for 1 h to facilitate cell adhesion. Firstly, the rat was sterilized using 75% alcohol, and the skin attached to the skull was carefully removed using clippers and forceps. Then, the skull was opened and the intact brain was isolated, ensuring no damage to the brain tissue. After this, the brain was quickly placed in a flat dish containing D-Hank’s balanced salt solution and placed on an ice tray, and the hippocampal neurons were isolated under a microscope. The isolated hippocampal neurons were digested in 0.125% trypsin at 37 °C for 15 min and then dispersed by gentle pipetting to obtain a cell suspension. Then, the hippocampal neurons were diluted and counted to achieve a cell density of 2.1 × $10^{6}$ cells/mL. Subsequently, 50 µL of the cell suspension was added to the center of each chip. After incubation for 1 h, 1 mL of complete culture medium (neurobasal medium (Gibco, Waltham, MA, USA) + 2% B27 (Gibco) + 1% P/S (Gibco) + 0.5% GlutaMAX (Gibco)) was added to the chip. Finally, the chips were transferred to a 37 °C, 5% CO_2_ incubator for cultivation. After 24 h, 50% of the culture medium was replaced, and, thereafter, the medium was changed three times per week, with 50% of the medium replaced each time.

In this study, the in vitro BNNs were recorded and stimulated by the MaxOne high-density microelectrode array system (MaxOne, Maxwell Biosystems, Zurich, Switzerland), as shown in Figure 2. The system comprises 26,400 recording electrodes and 32 stimulation channels that can be spatially configured in any arrangement. Each electrode measures 7.5 × 7.5 µm^2^, with inter-electrode spacing of 17.5 µm. The system is capable of simultaneously recording neural activity from up to 1024 channels.

Here, we selected 1024 electrodes with high spontaneous activity as the recording electrodes. The selection rule was as follows. Raw voltage signals detected by the MaxOne recording unit were filtered using a bandpass filter (300–3000 Hz), and a spike event was identified when the filtered voltage exceeded a threshold of 5.5 times the standard deviation of the background noise [23]. On the basis of the peak voltage of spontaneous spikes detected by each electrode, 1024 electrodes with the highest spike amplitudes were selected as recording electrodes for the experiments.

Among the selected 1024 electrodes, 8 electrodes exhibiting the highest spike amplitudes were designated as stimulation electrodes to ensure optimal stimulation efficiency. In accordance with previous studies [23], this study employed biphasic voltage pulses with an amplitude of 500 mV and a phase duration of 500 µs (positive phase followed by negative phase) as the fundamental units of the pulse stimulation sequences, which were used as input signals for the stimulation of the in vitro BNNs. The predefined pulse sequences were transmitted to the system hub via the Maxwell Python (3.13.2) application programming interface, where they were converted into sets of electrical stimulation commands for execution by the recording unit.

### 2.2. Visual Information Encoding and Decoding Methods

#### 2.2.1. Improved Delayed Phase Encoding

**Figure 3:**
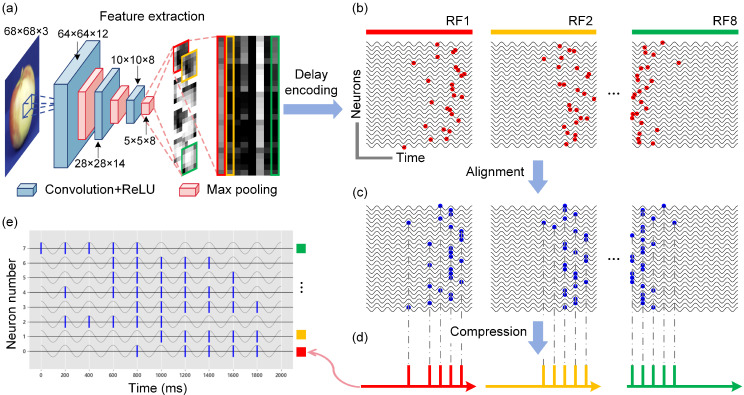
Framework of improved delayed phase encoding. (**a**) Feature extraction via CNN. (**b**) Conversion of features to pulse firing times. (**c**) Alignment operation. (**d**) Compression operation. (**e**) The resulting pulse sequences.

The proposed improved delayed phase encoding framework is shown in Figure 3. Delayed phase encoding is a classical method for the conversion of visual images into pulse sequences. However, the inability to handle high-dimensional color images and the high stimulation frequency of the encoded pulse sequences render this method inapplicable to in vitro BNNs [33]. To overcome these problems, in this study, we extracted features from images, performed temporal alignment and compression, and then transformed the processed data into stimulus inputs for in vitro BNNs. By discarding redundant image information, the number of required neurons is reduced while retaining the key features of the images, thereby enhancing the encoding efficiency.

This method employs a convolutional neural network (CNN) for image feature extraction before delayed phase encoding, generating feature maps with fewer pixels (Figure 3a). The designed CNN model consists of three convolutional layers, each followed by a max pooling operation, resulting in a six-layer feature extractor. The convolutional layers use filter sizes of 5 × 5 with stride of 1 and zero padding. All convolutional layers are followed by rectified linear unit (ReLU) activation functions to introduce non-linearity. The formula for the ReLU function is as follows:

(1) $$ReLU\left(x\right)=\left\{\begin{array}{cc} x\; & \;\mathrm{if}\;x>0\\ 0\; & \;\mathrm{if}\;x\leq 0 \end{array}\right.$$

Specifically, the number of filters in the three convolutional layers is set to 12, 14, and 8, respectively, progressively increasing the representation capacity of the model. The pooling layers utilize 2 × 2 max pooling with stride of 2, effectively halving the width and height of the feature maps at each stage. To train the CNN parameters, we selected 300 images from the ETH-80 dataset—100 images each of apples, cars, and cups. For each category, 70 images were randomly assigned to the training set, 10 images were used for validation, and the remaining 20 images were reserved for testing. We configured the learning rate to 0.0005, using the Adam optimizer and the cross-entropy loss function. The training was conducted for up to 50 epochs with the early stopping strategy based on the validation loss. The trained CNN serves as a fixed feature extractor that processes the input images into eight 5 × 5 normalized feature maps, which are subsequently fed into the delayed phase encoding module (Figure 3a). Each pixel unit in the feature maps generates precise firing timestamps through a defined spike-timing function:

(2) $$t_{i}=f\left(p_{i}\right)=t_{\max}\left(1-\arctan\left(\delta p_{i}\right)\right)$$

where $t_{i}$ is the firing time, $p_{i}$ is the normalized value of the pixel unit in the feature map, $t_{\max}$ is the maximum duration of the pulse stimulus, and δ is an adjustment parameter. Each feature map corresponds to a receptive field (RF), and the firing timestamps of the pixel units within the same RF are arranged into a time series (Figure 3b). By introducing a time offset, a temporal difference is subsequently created in the pulse firing times, which is calculated as follows:

(3) $$\Delta t_{i}=\frac{1}{n_{rf}}*i$$

where *i* is the position coefficient of the pixel point, and $n_{rf}$ is the size of the RF. In the traditional method, the pixel units in each RF have different sub-threshold membrane oscillation (SMO) functions. In order to compress the number of pulses, in this study, the pixel units in the same RF are set as SMO functions with the same initial phase:

(4) $$SMO=A\cos(\omega t+\varphi_{0})$$

where *A* is the amplitude of the subthreshold membrane oscillation, ω is the number of cycles of the oscillation, and $\varphi_{0}$ is the initial phase value, which is typically set to 0. Pulses at different locations are aligned to the nearest peak in the oscillatory function by means of the SMO function (Figure 3c). The time series, after phase alignment, are ultimately integrated by the neuron stimulation nodes corresponding to each RF and compressed into a series of pulse sequences (Figure 3d). Consequently, the original image, following the improved delayed phase encoding, produces 8 pulse sequences corresponding to the 8 selected stimulation electrodes (Figure 3e). Each pulse sequence has a stimulation duration of 2 s, with a minimum interval of 200 ms between adjacent pulses to ensure that the in vitro BNN recovers to its resting state before the next stimulation.

To measure the degree of difference between pulse sequences encoded by different images, each pulse sequence is mapped to an 8 × 10 binary matrix, where an element value of 1 indicates the presence of a pulse stimulus at the corresponding position, and a value of 0 indicates the absence of a pulse stimulus. The degree of difference between two pulse sequences is then quantified by calculating the Euclidean distance between the two matrices. A smaller distance indicates greater similarity in the encoding results, whereas a larger distance suggests a significant difference in the pulse sequences. The calculation formula is as follows:

(5) $$d=\sqrt{\sum_{i=1}^{m}\sum_{j=1}^{n}{\left(U_{ij}-V_{ij}\right)}^{2}}$$

where *U* and *V* denote two binary matrices of *m* rows and *n* columns.

#### 2.2.2. Logistic Regression Decoding

In this study, the decoding process employed a logistic regression model, which was trained by extracting feature vectors from the neural network’s firing activity. During the 2-second evoked firing signal segment recorded for each image stimulus, the firing signals detected within 10 ms after stimulation are discarded to avoid the influence of stimulation artifacts [9]. The signals are then downsampled, with each recorded electrode’s signal segment divided into 10 equal-length time windows (200 ms each). The number of neuronal firings within each time window is computed and combined into a feature sequence. Finally, the downsampled feature sequences from all recording electrodes are concatenated into a single feature vector. To prevent excessive feature vector dimensionality leading to model overfitting, principal component analysis (PCA) is applied to reduce the dimensionality of the vector, which is then input into the logistic regression algorithm for decoding. The logistic regression model recognizes input images by estimating the probability that a feature vector matches the firing pattern evoked by image-encoded pulse stimuli. This probability is calculated as follows:

(6) $$P\left(y=j|X\right)=\frac{e^{X\cdot \beta_{j}}}{1+\sum_{i=1}^{k-1}e^{X\cdot \beta_{j}}},j=1,\ldots ,k-1$$

(7) $$P\left(y=k|X\right)=\frac{1}{1+\sum_{i=1}^{k-1}e^{X\cdot \beta_{j}}}$$

where *X* is the feature vector; $P\left(y=k|X\right)$ is the probability that the feature vector belongs to evoked firing pattern *j*, where j=1,…,k−1; $\beta_{j}$ is the vector of regression coefficients for the evoked firing pattern *j*; and the pattern *k* serves as the reference pattern, whose regression coefficients are not computed directly but are derived from the probability supplements of other evoked firing patterns. The regression coefficient vectors are estimated using MATLAB’s (R2023b) mnrfit function, which employs the iteratively reweighted least squares algorithm to maximize the likelihood, equivalent to minimizing the cross-entropy loss function:

(8) $$L\left(\beta \right)=-\frac{1}{N}\sum_{i=1}^{N}\sum_{j=1}^{K}a_{ij}\ln\hat{P}\left(y_{i}=j|X_{i}\right)$$

where $a_{ij}$ is the indicator variable (1 if sample *i* belongs to category *j*, 0 otherwise). $\hat{P}\left(y_{i}=j|X_{i}\right)$ is the predicted probability. *N* and *K* represent the total numbers of samples and categories, respectively.

### 2.3. Network Functional Connectivity Analysis

The functional connectivity of in vitro BNNs refers to the temporal correlation of the firing activity across spatially distinct regions [34]. In this study, we recorded spontaneous firing signals from BNNs over a duration of 5 min using multiple recording electrodes. The spike time tiling coefficient (STTC) was employed to quantify the correlations between firing sequences captured by different electrodes, thereby assessing the strength of the functional connectivity between the nodes in the neural network [35]. The STTC correlation between the firing sequences $E_{1}$ and $E_{2}$ detected by two electrodes is calculated as follows:

(9) $$STTC\left(E_{1},E_{2}\right)=\frac{1}{2}\left(\frac{P_{E_{1}}-T_{E_{2}}}{1-P_{E_{1}}T_{E_{2}}}+\frac{P_{E_{2}}-T_{E_{1}}}{1-P_{E_{2}}T_{E_{1}}}\right)$$

where $T_{E_{1}}$ denotes the proportion of the total recording time that lies within ±Δt of any spike from $E_{1}$, and $P_{E_{1}}$ denotes the proportion of spikes from $E_{1}$ that lie within ±Δt of any spike from $E_{2}$. $T_{E_{2}}$ and $P_{E_{2}}$ are calculated similarly. The relevant time window (Δt) is set to 10 ms [36].

Given that the number of recorded electrode channels can reach 1024, computing the correlation matrix for all channels and generating a functional connectivity network involves high computational complexity and potential redundancy due to the proximity of adjacent electrodes. To address this, we employed the K-means clustering method for channel selection, which effectively reduces the computational complexity while preserving the spatial distribution characteristics of neural signals. Ultimately, the 40 channels closest to each cluster center were selected as representative channels. The adjacency matrix was used to display the STTC coefficients representing significant functional connections (edges) between the representative electrode channels (nodes). By integrating the physical locations of the electrode channels, we generated the functional connectivity network graph.

To analyze the characteristics of the network quantitatively, we calculated network metrics such as the node degree, node strength, and edge weight. The node degree refers to the number of edges connecting a node to other nodes. The edge weight represents the strength of the connection between two nodes [37]. The node strength is defined as the sum of the edge weights for a given node. The formulas are as follows.

Node degree:

(10) $$K_{i}=\sum_{j=1}^{N}A_{ij}$$

Edge weight:

(11) $$\omega_{ij}=STTC\left(i,j\right)$$

Node strength:

(12) $$S_{i}=\sum_{j=1}^{N}\omega_{ij}$$

where *A* is a binary quantity indicating whether node *i* and node *j* are connected in the adjacency matrix, and *N* is the total number of nodes in the network.

To investigate the dynamic changes in the internal structure of the BNNs, we employed a consensus clustering method to partition the neural network into a set of mutually independent functional modules [38]. Based on two parameters—the within-module degree z-score and the participation coefficient—we quantified the connectivity patterns of nodes within and between modules [39]. The formulas for these parameters are as follows.

Within-module degree z-score:

(13) $$z_{i}=\frac{k_{i}-{\bar{k}}_{S_{i}}}{\sigma_{k_{S_{i}}}}$$

where $k_{i}$ is the number of connections between node *i* and other nodes in its module $S_{i}$; ${\bar{k}}_{S_{i}}$ is the mean value of $k_{i}$ on all nodes in $S_{i}$; and $\sigma_{k_{S_{i}}}$ is the standard deviation of $k_{i}$ in $S_{i}$. The within-module degree z-score measures the relative degree of connectivity of a node in the module to which it belongs, and it is used to assess the importance of a node within a module.

Participation coefficient:

(14) $$P_{i}=1-\sum_{s=1}^{N_{m}}{\left(\frac{K_{iS}}{K_{i}}\right)}^{2}$$

where $K_{iS}$ is the number of connections from node *i* to the nodes in module *S*, $K_{i}$ is the total degree of node *i*, and $N_{m}$ is the total number of network modules. The participation coefficient is close to 1 if the node’s connections are evenly distributed across all modules, and it is close to 0 if all connections are in their own modules. The data for these metrics were visualized in the form of box-and-line plots, which were used to show the changes in the distribution of the data before and after training. Significant differences in the values of the characteristics before and after training were assessed by independent-sample *t*-tests and were considered statistically significant at *p* < 0.05.

The code is available in GitHub via https://github.com/wangxingchen231/Biomimetic-Visual-Information-Spatiotemporal-Encoding-Method, accessed on 29 May 2025.

## 3. Experiment and Results

**Figure 4:**
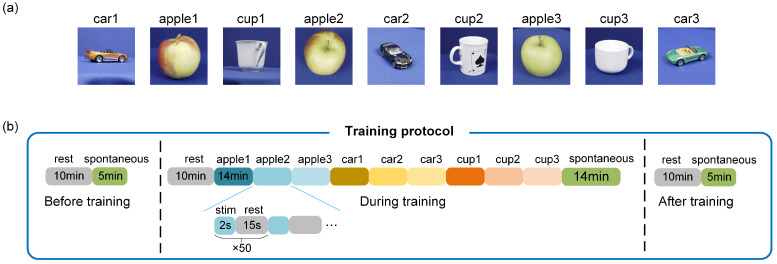
Experimental input images and training protocol. (**a**) A total of 9 images used in the experiment. (**b**) Training protocol.

To evaluate the image recognition performance of in vitro BNNs using the aforementioned encoding and decoding methods, and to explore how the stimulation paradigm affects network plasticity, this study conducted unsupervised training experiments on the BNNs through repeated stimulation. Previous studies have shown that successive repetitive stimulus inputs without applying feedback stimuli (unsupervised training) can effectively induce the unsupervised learning of in vitro BNNs [5,14,23,40] due to synaptic plasticity. Therefore, the same training method was used in this study. As illustrated in Figure 4a, the experiment utilized nine images from the ETH-80 dataset. During training, each of the nine images was encoded into a unique pulse sequence, and each sequence was delivered to the neural network 50 times as stimuli (Figure 4b). As described regarding the encoding method, each image stimulation lasted for 2 s, followed by a 15-second interval between stimulations to allow the neural network to return to its resting state. To enable the decoding model to differentiate between spontaneous firing and image-evoked firing, 50 segments of spontaneous firing activity—each lasting 2 s—were recorded under no-input conditions after the image stimulation. Spontaneous firing was detected for 5 min before and after training to assess the network functional connectivity. The effectiveness of the encoding and decoding methods was ultimately demonstrated by analyzing the response characteristics of the BNNs and the evolving trends in the network architecture.

### 3.1. High-Density Recording of Spontaneous and Evoked Activity in In Vitro Biological Neural Networks

**Figure 5:**
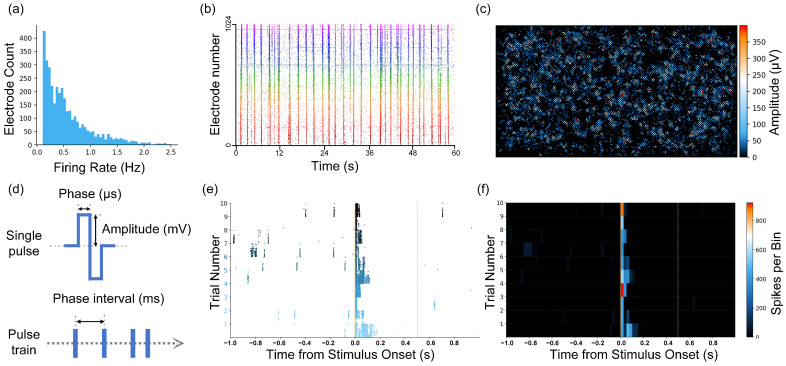
Spontaneous and evoked firing activity of in vitro biological neural networks. (**a**) Distribution of the spontaneous firing rate of the neural network. (**b**) Raster map of the synchronized bursting activity of the neural network. (**c**) Amplitude of neuronal firing detected in each channel. (**d**) Composition of the stimulation sequences and definition of the parameters. (**e**) Network activity evoked by stimulation of the 500 mV and 500 µs parameters (electrical stimulation was applied at 0 s). (**f**) Change in the firing rate o f the network after stimulation with the 500 mV and 500 µs parameters.

After 10 days of in vitro culture of the BNNs, we measured spontaneous firing using HD-MEAs that consisted of 26,400 electrodes. After this, electrical stimulation experiments were also conducted. At this stage, the spontaneous firing patterns of the neurons had reached a point of developmental stability, which was characterized by high-frequency spontaneous firing signals in the neural network (Figure 5a). Additionally, we observed spontaneous synchronized burst activity occurring at short time intervals (Figure 5b). To determine the locations of the active neurons, we detected the peak voltage amplitudes of spontaneous firings at each electrode (Figure 5c). This information was then used to select the recording and stimulation electrodes. As described in the Materials and Methods section, we employed biphasic voltage pulses with an amplitude of 500 mV and a phase duration of 500 µs (positive phase followed by negative phase) to ensure the magnitude and reliability of the evoked activity while minimizing neuronal damage (Figure 5d). With repeated stimulation using a single electrode, the in vitro BNNs generated stable evoked responses, with firing frequencies significantly higher than those seen in spontaneous firing (Figure 5e). The firing frequency gradually decreased within 0.2 s after stimulation, and the neural network returned to its resting state (Figure 5f).

### 3.2. Experimental Validation of the Visual Information Encoding Method for In Vitro Biological Neural Networks

**Figure 6:**
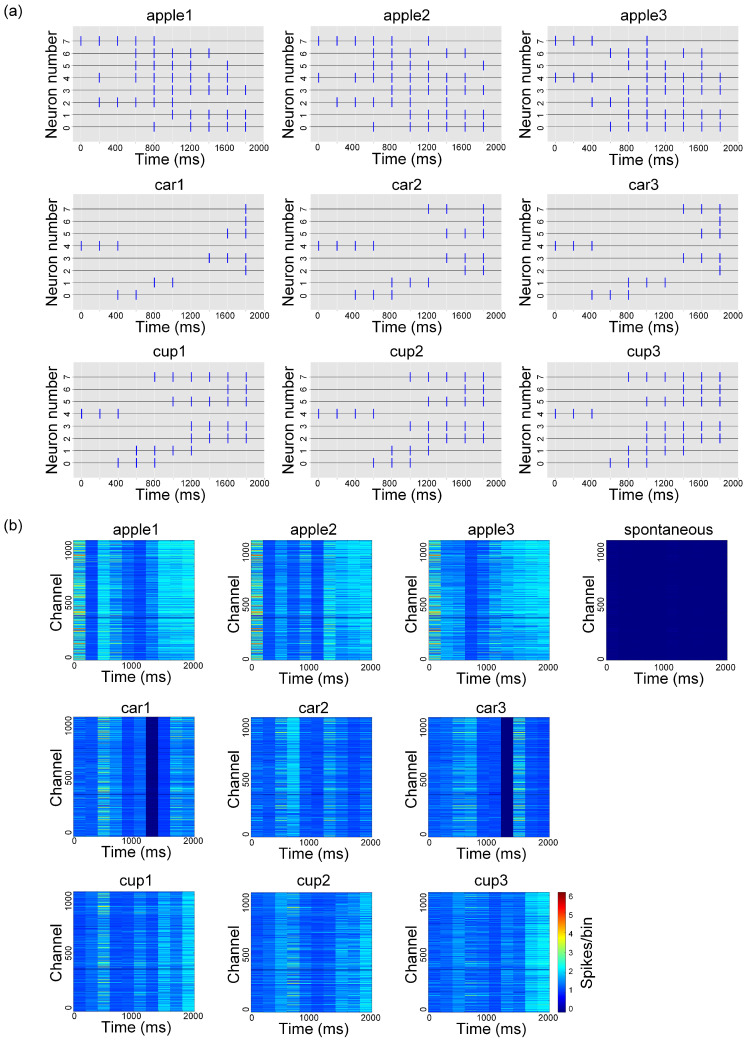
Image encoding results and evoked firing analysis. (**a**) Pulse sequences encoded from the 9 images. (**b**) Average evoked firing counts for the pulse sequence of each image.

This paper demonstrates the effectiveness of the proposed biomimetic visual information spatiotemporal encoding method by analyzing the differences in the pulse sequences for different image encodings and their corresponding average evoked firing patterns. Figure 6a displays the pulse sequences encoded in each image. Overall, different images are encoded to produce unique pulse sequences, while pulse sequences encoded from images with similar content exhibit similar distributional characteristics. Specifically, the pulse sequences encoded from apple images have the highest number of pulses, which are evenly distributed without significant temporal clustering. In contrast, the pulse sequences encoded from cup images show the distinct concentration of pulses in the later stages. Additionally, the pulse sequences encoded from car images contain the fewest pulses, exhibiting relatively sparse temporal and spatial distributions. Figure 6b illustrates the average evoked firing counts for the pulse sequences of each image. It shows that the pulse sequences encoded from apple images elicit noticeable evoked firing during the early stage (approximately within the 0–400 ms time window). These firings are distributed across multiple time windows, which aligns with the characteristics of their pulse sequences. The pulse sequences encoded from cup images induce denser evoked firings in time windows after approximately 1400 ms, resulting in a delayed response pattern. While the pulse sequences encoded from car images also elicit firings in the early-to-mid stage (approximately 400–800 ms) and later stages, the number of active channels involved is significantly lower than that seen for other pulse sequences, indicating lower network activity. In the absence of image input, no significant firing activity is observed in the BNNs. These results indicate that the number of pulses directly affects the neural network’s response magnitude: the more pulses present, the more neurons are activated, leading to greater network activity. Additionally, the evoked firing pattern is closely related to the distribution of the pulse sequences, with distinct image encodings triggering significantly different network firing patterns.

**Figure 7:**
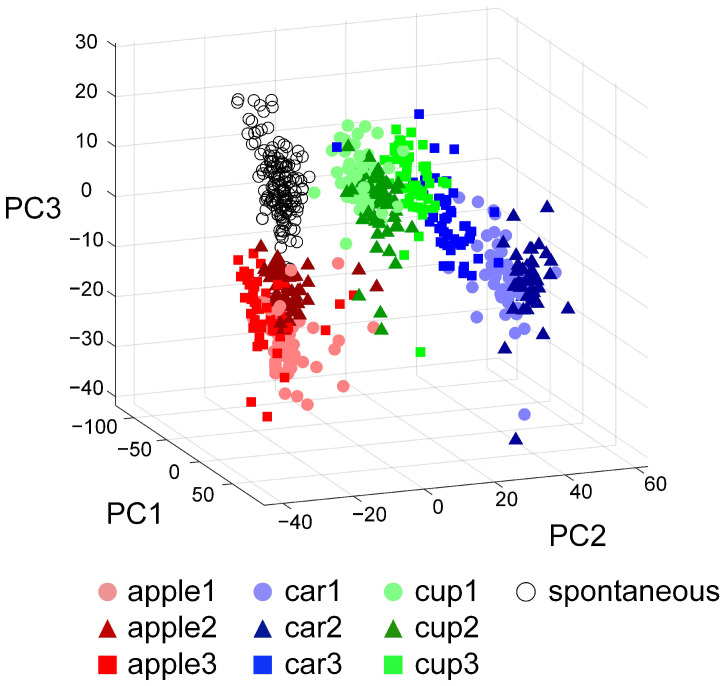
Characteristic distribution of image stimulus-evoked firing and spontaneous firing activity.

By utilizing PCA for the dimensionality reduction of the high-dimensional neural activity data, this paper further elucidates the distribution characteristics of the evoked firing patterns within the feature space in response to different image pulse stimuli. As shown in Figure 7, spontaneous firing patterns and stimulus-evoked evoked firing patterns are well separated along the PC1 axis, confirming the activation effect of external stimuli on BNNs. On the PC2 and PC3 planes, the evoked firing patterns corresponding to apple, car, and cup images form distinct clusters, demonstrating the separability of the neural responses to different image inputs. Moreover, the average Euclidean distance between the 0-1 matrices of pulse sequences encoded from car and cup images is 2.89 ± 0.48, which indicates the high similarity between the pulse sequences of these two types. Correspondingly, the distance between the distribution centroids of their evoked firing patterns in the feature space is 40.82, suggesting similar neural responses to both stimuli. In contrast, the average Euclidean distance between the 0–1 matrices of pulse sequences encoded from apple and car images is 4.74 ± 0.75, demonstrating a significant difference between the pulse sequences of these two types. The distance between the distribution centroids of the evoked firing patterns in the feature space is 62.91, suggesting that the neural responses to these two stimuli are quite different. We evaluated the BNNs’ ability to recognize similar images by computing the trace of the divergence matrix corresponding to the evoked response patterns of images with similar content. The divergence of the evoked firing patterns for all apple images is 2.68 × 10^4^, while that for all cup images is 2.15 × 10^4^, indicating that the BNNs have a limited ability to distinguish between different apples and different cups. Conversely, the divergence of the evoked firing patterns for all car images reaches 5.21 × 10^4^, markedly surpassing those of apples and cups, indicating that the BNNs are more effective in distinguishing different car images.

**Figure 8:**
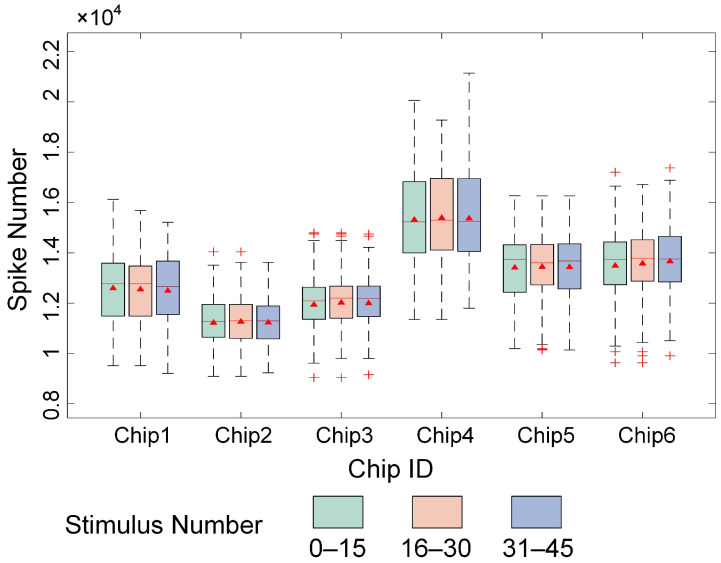
Number of evoked firings of the neural network under multiple stimuli.

This paper validates the stability of the encoding method and training strategy by analyzing the number of evoked firings in cultured BNNs during various stages of image stimulation. As shown in Figure 8, the 50 repeated stimulations of each image across six chips were divided into three training stages, i.e., 1–15, 16–30, and 31–45 stimulations, and the number of evoked firings in each step was statistically analyzed. The results demonstrate that, as the number of stimulations increased, the number of evoked firings across the six chips did not significantly change during the different steps. The firing rates remained stable across all steps, indicating that the designed encoding method generated pulse stimulation sequences capable of effectively maintaining normal neural firing dynamics in cultured BNNs over prolonged and repeated stimulation, without significant declines or overactivation.

### 3.3. Spatiotemporal Combined Stimulus Pattern Recognition and Learning Behavior in Biological Neural Networks

**Figure 9:**
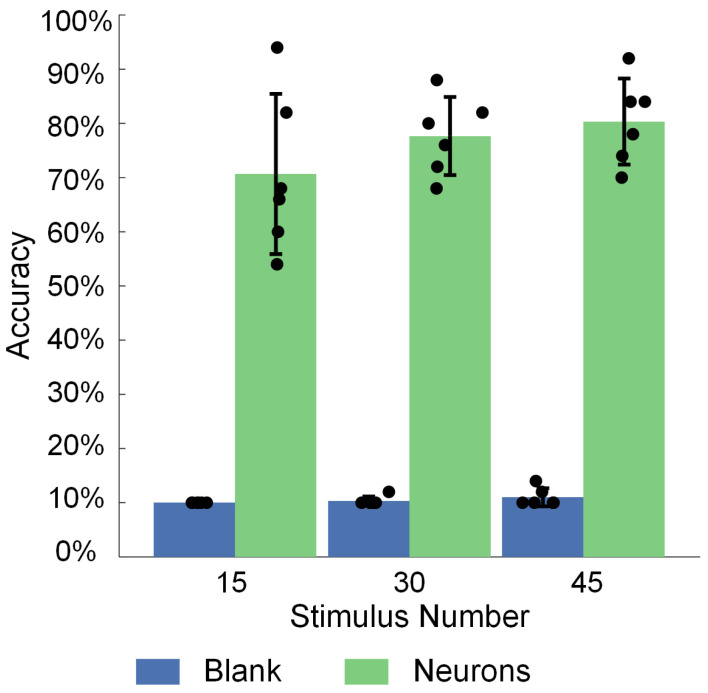
Variations in the neural network’s image recognition accuracy during the three stages of training.

By evaluating the image recognition accuracy of cultured BNNs across different training steps, we validate their ability to recognize and learn spatiotemporal combined stimulation patterns. In this study, nine image-evoked firings for each of the three stimulation steps (i.e., firings 1–15, 16–30, and 31–45) were combined with data from spontaneous neural network firings into ten firing patterns. The feature vectors extracted from the data of these ten discharge patterns were used to train the parameters of a logistic regression model in the decoding layer. Five firing data after each of the three stages were used to test the image recognition accuracy of the neural network for the corresponding stage. The results demonstrate that (Figure 9), under the condition of neuronal participation, the recognition accuracy increases from 70.67% ± 14.79% in the first step to 77.67% ± 7.20% in the second step and further to 80.33% ± 7.94% in the third step, representing an overall improvement of 13.64% in the recognition performance of the BNNs. In contrast, under the blank control condition (without cultured neurons), the recognition accuracy remains near random levels, with values of 10.00% ± 0.00%, 10.33% ± 0.82%, and 11.00% ± 1.67% across the three steps, confirming that the blank chips lacking neurons do not possess computational capabilities. These findings indicate that, with the accumulation of training, cultured BNNs gradually adapt to external inputs and increase their ability to recognize specific information.

**Figure 10:**
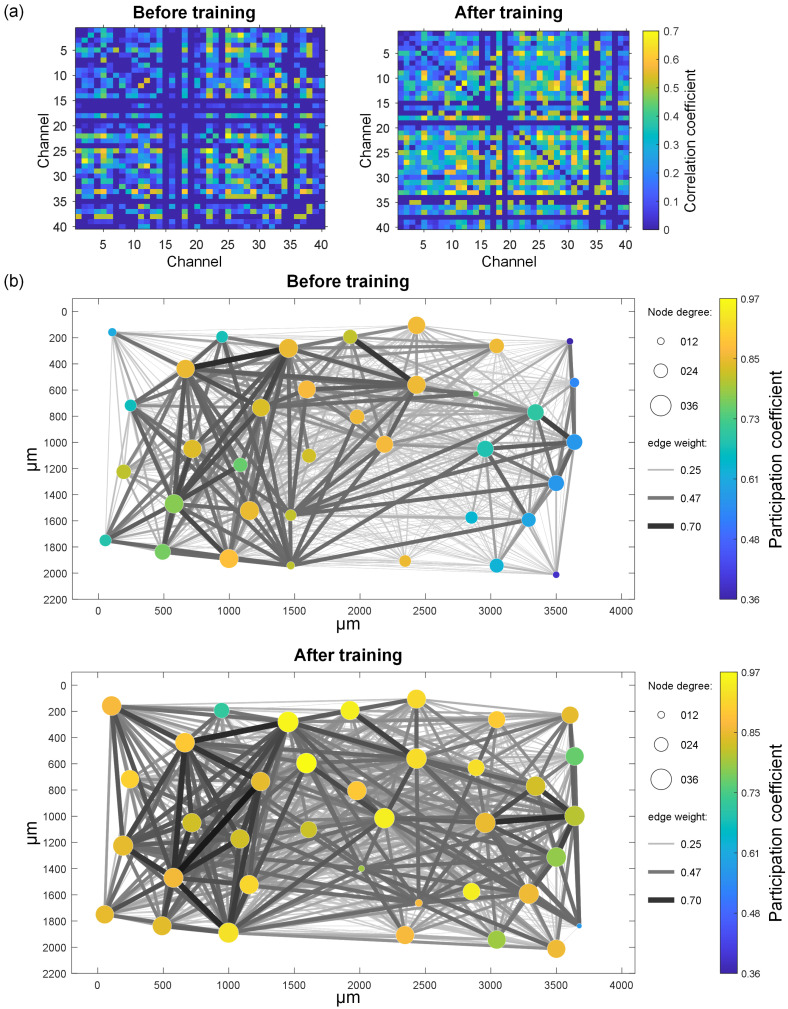
Comparison of neural network structures before and after training. (**a**) Correlation matrix representation of connection weights between representative electrodes before and after training. (**b**) Functional connectivity topology of the neural network before and after training.

By detecting the spontaneous firing of cultured BNNs before and after three-stage stimulation training and calculating the pairwise STTC correlations of 40 representative electrodes to quantify the synchronized firing relationships between neurons, this study further investigates the effects of electrical stimulation training on cultured BNNs. In Figure 10a, the correlation matrix calculated from the spontaneous firing activity of a representative chip before and after three-stage stimulation training is displayed. Before training, the electrode channels of the chip generally exhibit low correlation levels, with only a few channel pairs showing relatively strong correlations (e.g., channels 22 and 25), and the distribution of correlations is relatively scattered. After training, the correlation matrix becomes significantly denser, with an overall increase in the correlation coefficients, particularly in specific regions where strong correlations are observed (e.g., channels 22 to 33). Figure 10b further illustrates the spatial dynamics of the functional connectivity in the neural network before and after training through a visualized functional connectivity network. Before training, the network structure is sparse, with fewer and weaker connections between nodes, which are primarily concentrated in the left region. The node degree and participation coefficient exhibit significant heterogeneity, indicating that the network has not yet formed efficient information transmission pathways. After training, the network structure undergoes significant changes, becoming more uniform. Previously unconnected nodes form new pathways, the number of connections between nodes increases, and the weights of connections, especially at key nodes, are significantly increased. These structural changes suggest that the neural network gradually optimizes its topological structure through external stimulation training, enhancing its ability to integrate and transmit information.

**Figure 11:**
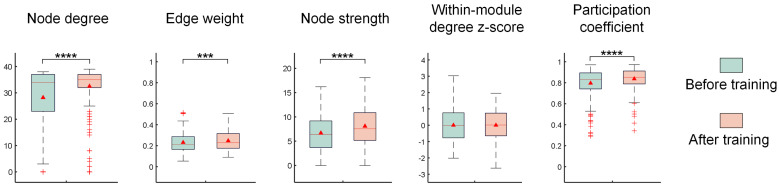
Statistical analysis of key metrics of neural networks before and after training (significance level: *** *p* < 0.001, **** *p* < 0.0001).

This study further validates the optimization effect of training on the topological structure of the network by statistically analyzing network characteristic metrics across six chips, revealing trends in functional connectivity changes. As shown in Figure 11, after training, the node degree and edge weight significantly increase from 28.20 ± 11.82 and 0.23 ± 0.09 to 32.49 ± 8.55 (*p* < 0.0001) and 0.25 ± 0.10 (*p* < 0.001), respectively, indicating that the training process not only increases the number of connections but also enhances their strength. The node strength increases from 6.66 ± 4.12 to 8.08 ± 4.08 (*p* < 0.0001), reflecting a significant improvement in the information transmission capacity of network nodes. The within-module connectivity measures the deviation of a node’s degree from the average degree of nodes within its module. Higher within-module connectivity indicates that the node is closer to being a “central node” within its module. The participation coefficient measures the distribution of a node’s connections across different modules. A value close to 1 indicates that the node’s connections are evenly distributed across all modules, serving as a bridge between modules, whereas a value close to 0 indicates that the node’s connections are almost entirely concentrated within its own module, with few connections to other modules. Under the experimental conditions of this study, the participation coefficient across the six chips significantly increases from 0.80 ± 0.14 to 0.84 ± 0.10 (*p* < 0.0001) after training, while the within-module connectivity does not change significantly. This finding suggests that connections within the same module remain relatively uniform and that the internal structure of the modules remains stable after stimulation. Moreover, network nodes form more connections with other modules, and these connections are more evenly distributed across different modules.

## 4. Discussion

This study proposes a biomimetic visual information spatiotemporal encoding method for in vitro BNNs and validates the feasibility of using the electrophysiological properties of these networks to achieve image perception and recognition under external stimulation. The experimental results show that different image stimuli evoke specific response patterns in cultured BNNs, indicating that BNNs have the ability to recognize multiple types of spatiotemporal stimulation patterns. Additionally, the evoked response patterns for each image are highly correlated with the distribution of pulse sequences, suggesting that spike distribution features are a core factor driving the BNN’s ability to recognize images. Furthermore, existing research has demonstrated the intelligent behaviors of cultured BNNs in specific tasks, such as obstacle avoidance in mobile robots [19], playing the arcade game `Pong’ [22], speech recognition [23], and dexterous hand joint movement control [24]. As for image recognition, previous studies have used a spatial encoding method that maps the pixels of simple character images to electrodes at corresponding positions on a microelectrode array [40,41]. It applies a stimulus to the corresponding electrode only if the pixel is part of the constituent pattern. However, due to its inability to convert images into temporally structured pulse sequences, this method is limited to encoding binary and low-resolution images that can be represented using only a few electrodes. In comparison, the proposed method addresses the limitations of cultured BNNs in perceiving high-dimensional image information, expanding their application boundaries in the field of image processing. Meanwhile, our method is capable of handling high-resolution images with greater robustness. This study lays the foundation for neuro-robotic systems based on cultured BNNs to achieve complex environmental information perception.

This study confirms that spatiotemporal combined stimulation can induce learning behavior in cultured BNNs through an unsupervised training strategy. A notable characteristic of cultured BNNs is their network plasticity, which endows them with a high capacity for learning. Previous research has shown that applying low-frequency (0.02–1 Hz) [42,43] or high-frequency (20 Hz) [44,45] electrical stimulation with fixed parameters to single or multiple electrodes can effectively induce learning and memory functions in cultured BNNs. In contrast, this study employed a set of pulse sequences encoded from images as stimulation signals, characterized by dynamic changes in both the electrode combinations and timing, with a maximum stimulation frequency of 5 Hz. Under this spatiotemporal combined encoding paradigm, the neural network transitions from learning on the basis of single spatial stimuli to adapting to and learning from combinations of multiple spatial stimuli across continuous time windows. The experimental results demonstrate that, as the number of training steps increases, the image recognition accuracy of the neural network gradually improves. It sufficiently indicates that the network can adapt to complex spatiotemporal stimulation patterns and possesses basic unsupervised learning capabilities.

Furthermore, by comparing the functional connectivity before and after training, it is evident that unsupervised training significantly enhances the network’s functional connectivity, making the network more tightly integrated and coordinated. These structural changes may result from the network’s long-term adaptation to external input, further validating its strong learning capacity. The significant increases in the node degree, node strength, and edge weight suggest that the network can reshape neural connections during learning, thereby improving the efficiency and accuracy of information transmission. This conclusion aligns with previous findings suggesting that training induces substantial modifications in the structural and functional connectivity of neural networks—including the strengthening of synaptic links, emergence of new pathways, and enhanced global integration—all of which contribute to improved pattern recognition performance [23,40]. The modularity analysis further reveals the organizational structure and hierarchical characteristics of the network. After training, the within-module connectivity of the network nodes does not change significantly, whereas the participation coefficient between modules increases notably. This finding indicates that, after repeated stimulation, the connection patterns within modules remain relatively stable, whereas the connections between modules are strengthened. This reflects the network’s enhanced ability to utilize information from different modules, improving its recognition performance for input data. Such cross-module connections may facilitate more comprehensive feature extraction and pattern recognition when the network encounters diverse inputs. This finding aligns with the “small-world network” characteristics observed in biological neural systems, where enhanced local connections and inter-module integration enable efficient information transmission while minimizing energy consumption [46].

Previous studies have also shown that trained BNNs typically exhibit stronger evoked responses under the same electrical stimulation [47]. However, in the three training steps of this experiment, no significant enhancement in the evoked responses was observed. This result highlights the high complexity and diversity of the effects of electrical stimulation on neural network plasticity [48]. Different stimulation patterns and training strategies may guide the network toward different directions of plasticity, and changes in a single feature are insufficient to fully reveal the dynamic processes of learning mechanisms and functional remodeling in neural networks. Therefore, a multi-dimensional and multi-level comprehensive analysis is necessary to more thoroughly understand the adaptation and learning mechanisms of BNNs in response to external stimuli.

BNNs show specific characteristics. BNNs exhibit inherent nonlinear computational abilities and network plasticity, allowing for complex information processing, real-time learning, and memory functions. Moreover, BNNs transmit information through electrochemical signals, which offers significant advantages in terms of energy consumption. For instance, the human brain operates on approximately 20 watts of power, while an artificial neural network (ANN) of a similar size might consume several kilowatts [14]. Furthermore, BNNs have high learning efficiency. Research conducted by Cai et al. [23] has demonstrated that in vitro BNNs can reduce the training time by over 90% when compared to conventional ANNs, such as long short-term memory (LSTM) networks. However, BNNs are inherently difficult to culture and maintain, requiring precise environmental control and expert handling to ensure long-term viability and functionality.

The aim of this study was to develop a visual information encoding method that constructs an efficient pathway for visual information perception, specifically for in vitro BNNs. The study verified the effectiveness of the proposed encoding method by accurately recognizing pulse sequence stimulus patterns encoded from nine images. The introduction of the feature extractor theoretically enables the proposed encoding–decoding framework to be applicable to the perception of any complex visual scene. However, this study only evaluated the recognition performance of BNNs on a relatively small image dataset, which did not fully utilize the computational and learning advantages of BNNs. In our future work, we plan to conduct experimental tests using image datasets that include a greater variety and larger volume of data to further validate the advantages of BNNs and explore their computational limits. Our current proposed encoding method focuses on static image inputs and thus the resulting in vitro BNNs are still unable to process or recognize dynamic visual information such as videos. However, BNNs have a natural temporal encoding mechanism to capture temporal information as pulses in dynamic signals, allowing them to process visual sequence data with rich temporal dependencies. Thus, in the future, we will investigate the possibility of decomposing dynamic images into multiple static frames to create a time series and then apply it to the in vitro BNNs for tasks such as action recognition [49,50] to evaluate its performance in processing dynamic visual information.

## 5. Conclusions

We have proposed a biomimetic visual information spatiotemporal encoding method for in vitro biological neural networks (BNNs). This method transforms high-dimensional images into low-dimensional electrical stimulation pulse sequences that are suitable for these networks. We conducted unsupervised training on cultured hippocampal neural networks using high-density microelectrode arrays (HD-MEAs), enabling the networks to learn to improve their computational performance and accomplish image recognition tasks. Activity information from in vitro BNNs was decoded by a logistic regression model. Through the analysis of multiple image-evoked response features, we validated the feasibility of in vitro BNNs, achieving high-dimensional image perception via electrophysiological properties under external stimulation. Additionally, on the basis of the correlation of the network node firing sequences, we constructed functional connectivity maps of the in vitro BNNs before and after training, revealing the significant impact of spatiotemporal combined encoding-based external stimulation training on neural network functional connectivity. The experimental paradigm and results reveal the potential of integrating in vitro BNNs with robotic systems to complete intelligent tasks and provide important evidence for an understanding of the plasticity mechanisms of neural networks.

## Data Availability

The raw data supporting the conclusions of this article will be made available by the authors on request.

## Abbreviations

The following abbreviations are used in this manuscript:

BNN	Biological neural network
HD-MEA	High-density microelectrode array
CNN	Convolutional neural network
ReLU	Rectified linear unit
RF	Receptive field
SMO	Sub-threshold membrane oscillation
PCA	Principal component analysis
STTC	Spike time tiling coefficient
ANN	Artificial neural network
LSTM	Long short-term memory
